# CT Imaging in Neurocritical Care

**DOI:** 10.5005/jp-journals-10071-23185

**Published:** 2019-06

**Authors:** Rajat Bhargava

**Affiliations:** Department of Neurocritical Care and Anesthesia, Fortis Mulund, Mumbai, Maharashtra, India

**Keywords:** Calcification, Hemorrhage, Sulci

## Abstract

CT imaging is the ideal technique for initial evaluation of neurocritical care patients. It is robust, reliable and relatively easy to interpret. Its ability to diagnose hemorrhage, bony fractures, brain edema and herniation in short scanning times makes it an ideal technique for neurological emergencies. The imaging equipment, normal anatomy and imaging findings in pathologies like infarct, hemorrhage, trauma and hydrocephalus are reviewed in the article.

**How to cite this article:**

Bhargava R. CT Imaging in Neurocritical Care. Indian J Crit Care Med 2019;23(Suppl 2):S98–S103.

## INTRODUCTION

Computed Tomography (CT) scan is an ideal imaging technique for initial evaluation of neurocritical care patients. CT imaging is robust, reliable and easy to perform and even the non-radiologist clinicians are able to interpret the imaging findings. The short scanning times and its widespread availability also makes it an attractive imaging tool contributing to its universal use in these subgroup of patients. These strengths and the utility of CT scanners have prompted several big hospitals to install portable CT scanners in the neuro ICU's which have proven effective in improving care of the patients. CT images show both the soft tissue brain parenchyma and bone equally well. The ability to image bony fractures in the cranium and in the spine is of immense value in neuro trauma patients. CT scan also permits performance of contrast enhanced angiography which plays an important role in acute stroke and subarachnoid hemorrhage settings.^1^

## EQUIPMENT

Most of the imaging equipment available in secondary and tertiary care hospitals are now multidetector CT scanners (MDCT) which enable high quality imaging in a short span of time. MDCT scanner which is 16 slices and above is ideal for imaging neurocritical care patients. One can also perform angiography, perfusion CT scans on these MDCT scanners. Multidetector CT scans permit sub millimeter thin slices of brain which can then be reconstructed in multiple planes to depict the anatomy to the surgeons in more physiological manner. Portable CT scanners placed in the neuro ICU are new development proving to be of immense benefit care of patient. They are generally of 8 slices and above configuration allowing images of 1.25 mm minimum thickness. They have a small foot print gantry and patient can be scanned on the bed itself without need to transfer them on table. The image quality has been rated as excellent in several published studies. The biggest benefit of CT scanner placed in the ICU is a quick turn-around time while avoiding the risk of transferring an unstable patient to radiology department. However, cost remains a barrier in wide adoption of this technique in our country.^2^

## TECHNIQUE

For CT Brain, axial images are obtained covering from top of the head to the base skull including the orbits. The section thickness in mutislice CT is usually less than 1.0 mm. However, for imaging documentation 5 mm slices are reconstructed and printed. The radiation dose is usually less than 2 mSV. The images are seen in soft tissue window setting for brain parenchyma and after adjusting the contrast in bone windows setting as well. To assess fractures, images reconstructed using sharp bony algorithms should be looked at.

For most of the neurocritical situations like trauma and stroke, a plain non IV contrast CT scan is adequate. The IV contrast CT scans are indicated in looking for focal lesions, tumors of the brain and meningitis setting. However the use of contrast-enhanced CT scan (CECT) has largely been supplanted by MR imaging. MRI has far higher contrast resolution than CT scan and its ability to identify disease pathology from the normal brain parenchyma is superior to CT scan. However MRI takes significantly longer time, making it unsuitable in critical care settings. CT is also better than MRI in detecting hemorrhage, calcification and bony fractures.

## ANATOMY

**Fig. 1:**
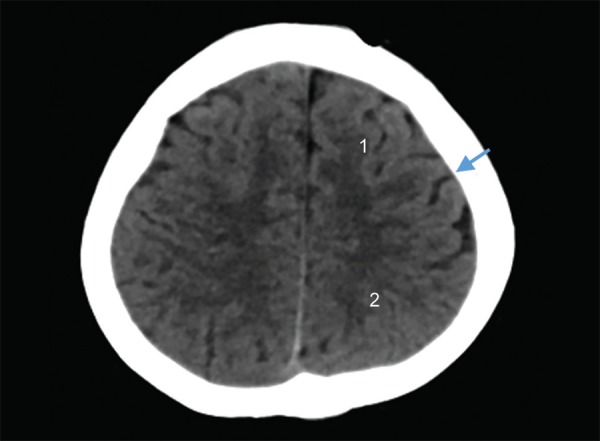
Normal anatomy: (1) Frontal lobe; (2) Parietal lobe, Arrow - central sulcus

**Fig. 2:**
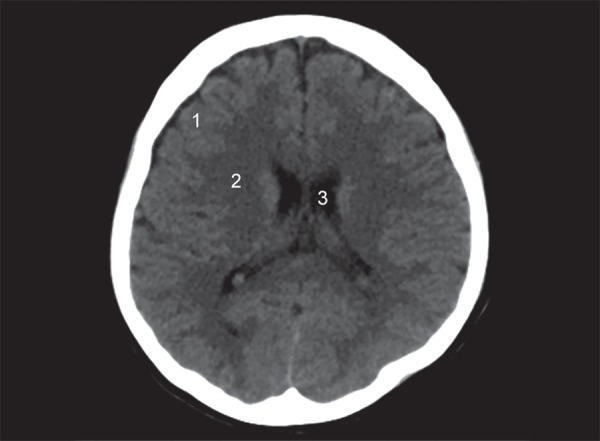
Normal anatomy: (1) Grey matter; (2) White matter; (3) Lateral ventricles

**Fig. 3:**
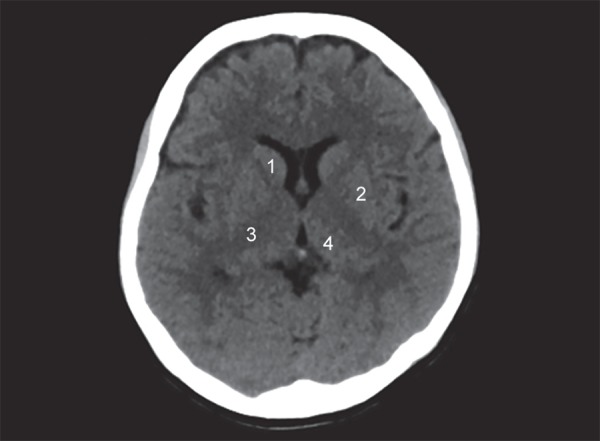
Normal anatomy: (1) Caudate nucleus; (2) Lentiform nucleus; (3) Internal capsule; (4) Thalamus

**Fig. 4:**
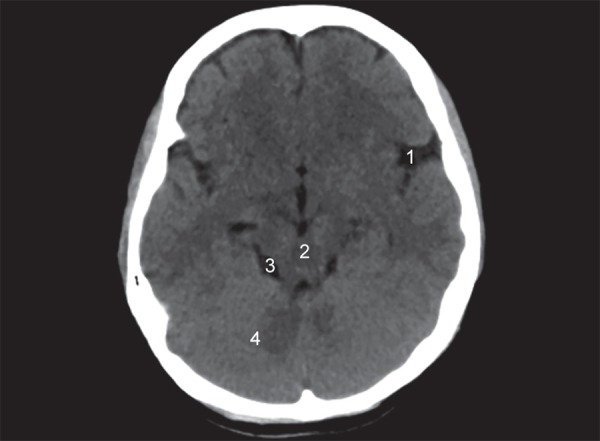
Normal anatomy: (1) Sylvian fissures; (2) Mid brain; (3) Basal cisterns; (4) Cerebellum

The lobes of the brain are separated by sulci. The central sulcus separates frontal from the parietal lobe (Fig. 1), the parieto occipital sulcus separates the parietal from the occipital lobe and the sylvian sulcus separates the temporal lobe from the rest of the cerebral hemisphere (Fig. 4). In brain imaging, assessment of symmetry of the left and right halves of the brain is important. The midline falx is a dural sleeve which separates the cerebral hemispheres into the left and right halves. The tentorium sleeve of the dura separates the cerebellum and brainstem from the supratentorial cerebral hemispheres. On CT images, the cortex comprising grey matter appears hyperdense compared to the white matter which appears hypodense (Fig. 2). The deep grey nuclei which include the basal ganglia, thalamus appear hyperdense, similar to the cortex (Fig. 3). The cerebrospinal fluid containing structures which include the ventricles and cisterns are hypodense in the appearance (Fig. 4).

## PATHOLOGIES

Most of the pathologies are identified either by change in the density of the normal brain structure or by focal mass effects/welling which is causing displacement and asymmetry of the intracranial contents. Change in the density can be both hypodense and hyperdense. Usually on CT scan, hyperdense shadow indicates hemorrhage, calcification. The measurement of Hounsefield units (HU) is a software which assigns numbers to the density of the structures on the CT image. It is calibrated in such manner that air which appears black has a HU value of −1000, fat which is dark is −100 to −50, water 0, white matter 20–30, grey matter 37–45, hemorrhage 45–65, soft calcification 60–80, dense calcification 200–400, bone 700–3000 and metal 4000.^2,3^

## INFARCT

**Fig. 5:**
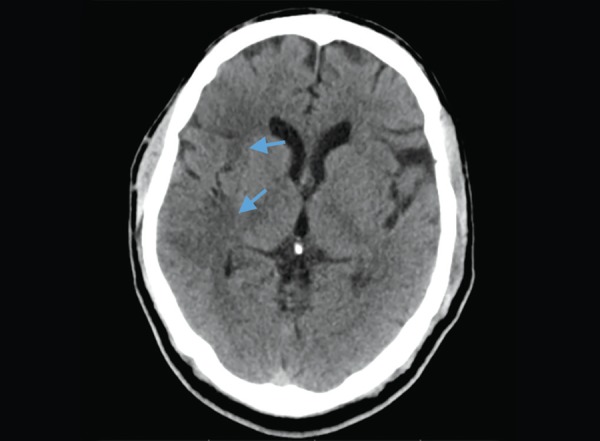
Hyperacute infarct: loss of cortical definition in right fronto-parietal lobes and insular cortex

**Fig. 6:**
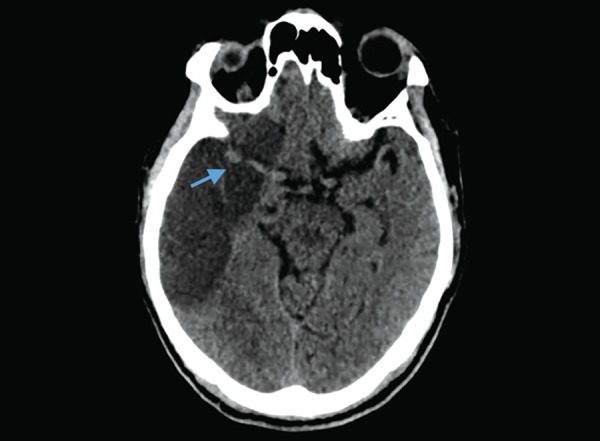
Right middle cerebral artery appears hyperdense (arrow), commonly referred as “Hyperdense MCA sign” suggests occlusion. Wedge shaped hypodense area in MCA territory suggests acute infarct

CT scan plays an invaluable role in diagnosing as well as deciding the treatment algorithms in acute brain infarction. CT scan is relatively insensitive in diagnosing hyperacute infarct in the first couple of hours. However on a closer look, few early signs of acute stroke can be appreciated. The earliest sign of acute infarct is a subtle loss of grey white matter difference, best appreciated as loss of insular ribbon (Fig. 5). Other useful signs include a subtle decrease in density of the basal ganglia and hyperdense vessel sign which represents an occluded and thrombosed artery (Fig. 6). Hyper dense MCA can be seen in the sylvian fissure in cases acute MCA territory infarcts. One of the main roles of CT scan in hyper acute stroke settings is to exclude hemorrhage. With a definite clinical diagnosis of stroke, absence of hemorrhage on CT scan enables the clinicians to proceed with thrombolysis in appropriate settings.

**Fig. 7:**
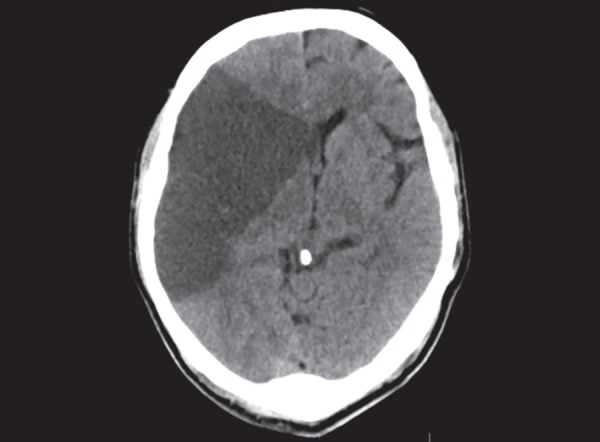
Right MCA territory acute infarct: well-defined hypodense area in frontotemporoparietal lobes, insular cortex, caudate nucleus and lentiform nucleus involving both cortex and white matter

Infarcts which are more than 6–8 hours old, generally start to appear on CT scan as hypodense, sharply demarcated wedge shaped areas involving both the grey and subcortical white matter. With temporal progression, the infarct becomes more hypodense and well-defined and starts to develop the mass effect (Fig. 7).

By 3rd day, the infarct develops maximum swelling and mass effect, which in cases of large large territory infarcts lead to midline shift and compression of the ventricles and cisterns. Some infarcts especially cardioembolic ones will show hemorrhagic transformation which appears as mild gyral hyperdensity. The hypodensity in infarcts also follows the vascular supply territory pattern. The infarct also progress over time, with the mature infarcts beyond 7 days starting to show loss of mass effect. A chronic infarct beyond 21 days will become gliotic, show volume loss with widening of the adjacent brain sulci. Subacute infarct will also show a pattern of gyral enhancement and if IV contrast is given in such situation, it can be misinterpreted as encephalitis.

Infarcts can also be lacunar in nature, secondary to thrombosis of the small penetrating vessels. These are seen as small hypodensities in the deep white matter or the grey matter nuclei, which can be initially difficult to appreciate. Chronic lacunar infarcts will become fluid density holes. A watershed territory infarct is seen as a wedge shape hypodensity located in the brain parenchyma overlapping the two vascular territories. These are seen either in the ACA- MCA watershed zones or the MCA- PCA watershed zones. Another watershed territory is between the penetrating vessels and superficial cortical vessels in which multiple small infarcts are seen in a vertical line involving deep frontoparietal white matter. Watershed infarcts are often associated with hypotension and carotid vascular stenosis.

Small vessel arteriosclerotic changes, mostly secondary to hypertension induces a multifocal and diffuse pattern of hypodensity mostly in the deep white matter and deep grey nuclei which represents chronic ischemic changes in the brain.

In the acute setting, CT angiography of the brain and neck vessels can be performed to assess for large vessel occlusion. A thrombus in the ICA or the M1 segment of MCA in the appropriate settings should be treated with mechanical thrombectomy. With the current guidelines, IV thrombolysis can be performed upto 4.5 hours of the onset of stroke and intra arterial mechanical thrombectomy is usually performed upto 6 hours after the onset. With the new published trials, the window for intra arterial thrombectomy can now be extended beyond 6 hours if clear documentation of salvageable brain can be documented on CT perfusion imaging.

## HEMORRHAGE

**Fig. 8:**
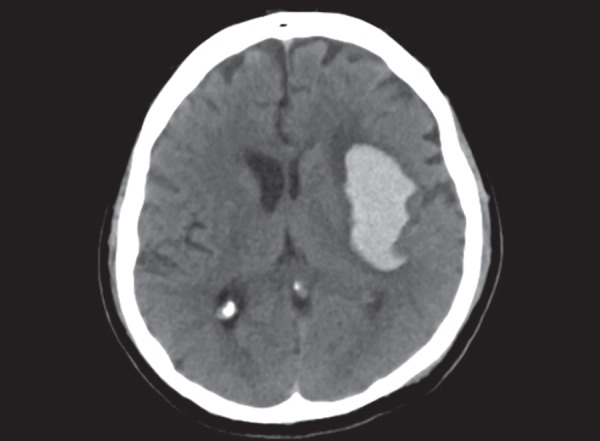
Hypertensive bleed: well-defined, hyperdense area in left lentiform nucleus and corona radiata. Note the mass effect on left lateral ventricle

The role of CT scan is unsurpassed in detecting acute intracranial hemorrhage. Intracranial hemorrhage can be parenchymal, intraventricular, subarachnoid and in the extra parenchymal spaces in relation of the dura. Acute hemorrhage appears a hyperdense on the CT scan (Fig. 8). The blood progressive gets more hyperdense as it clots over time. It remains hyperdense till approximately first 7 days, then progressively starts to lose its density. The hematoma starts to clear from the periphery first, becoming hypodense, while the central portion remains hyperdense. At 4 weeks time, the hematoma resolves appearing hypodense without any mass effect. Occasionally hemorrhage can be confused with contrast enhancement especially in patients who are imaged after intra-arterial thrombolysis. Dual energy CT done at both 80 and 140 Kv is useful in differentiating these two conditions. The iodine overlay maps show contrast enhancement as hyperdense while hemorrhage is not seen hyperdense thus separating the two.

**Fig. 9:**
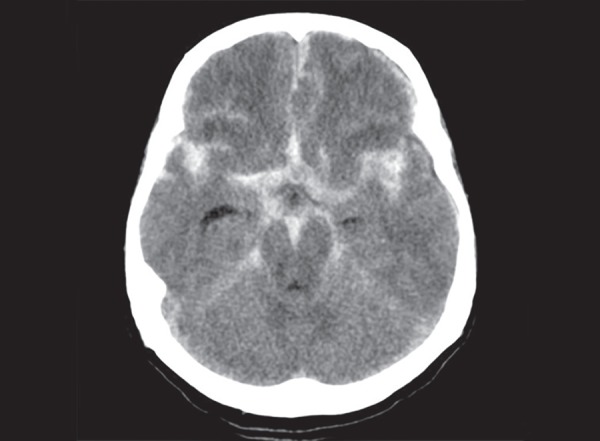
Subarachnoid hemorrhage: diffuse hyperdense areas in all basal cisterns and both Sylvian fissures

**Fig. 10:**
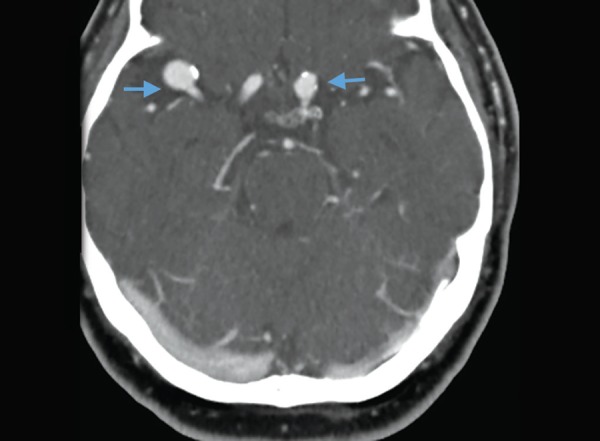
Aneurysms on CT angiography: two contrast filled out pouchings (arrows) along right MCA and left supraclinoid ICA

**Fig. 11:**
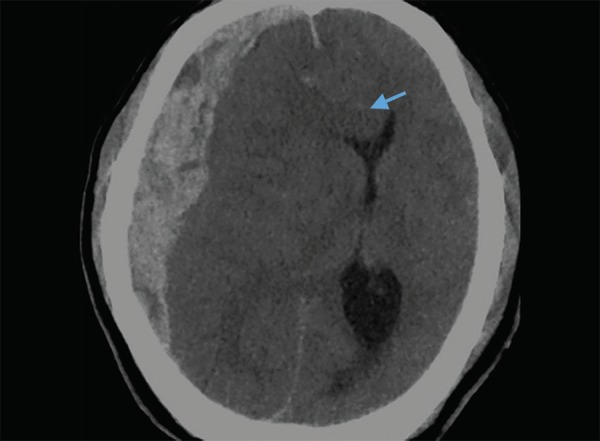
Acute subdural hematoma: extra-axial hyperdense area along calvarium of right frontoparietal regions. Note the midline shift, subfalcine herniation (arrow) and cerebral edema

Spontaneous parenchymal hematomas are mostly secondary to hypertensive bleed. The common sites for hypertensive bleed in order of frequency are gangliocapsular region, thalamus, pons, cerebellum and lastly lobar parenchyma (Fig. 8). Homogenous blood density collection in the deep grey nuclei region is likely secondary to hypertension and does not need further investigation to ascertain its cause. Frequently there will be an extension of thalamic hematoma into ventricles. Large intraventricular hemorrhage extension can lead to hydrocephalus and may require an intraventricular drain. Presence of lobar hematoma requires further investigation to ascertain its cause, common being an arteriovenous malformation. Diagnosis of AVM can be achieved with the CT angiography, MRI, however DSA will be needed for exact delineation of feeding arteries. On CT angiography, hypertrophied dilated branch of artery is seen with early filling and dilated drain veins. Parenchymal hematoma can also be secondary to bleed in a tumor. Tumoral bleed appears heterogenous with variable density of blood representing bleeding at different time events. The presence of an underlying tumor is well delineated with the contrast enhanced MRI.

In subarachnoid hemorrhage, there is hyperdense blood seen within the basal cisterns and also within the convexity sulci (Fig. 9). Subarachnoid hemorrhage is mostly secondary to vascular aneurysms. The cause of subarachnoid hemorrhage is best identified with contrast enhanced CT angiography. CT angiography requires a rapid injection of iodinated contrast though a large bore IV and rapid acquisition of thin slice CT scans. CT angiography is highly sensitive and specific for diagnosing intracranial aneurysm. The anatomy of the aneurysm is delineated in a 3D format enabling the surgeon or the interventional radiologist to plan the treatment (Fig. 10).

**Fig. 12:**
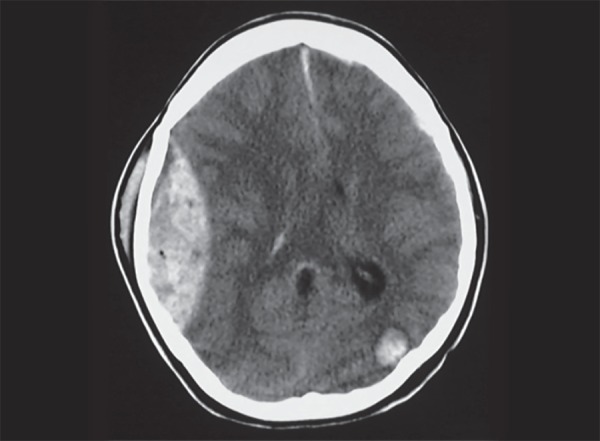
Acute extradural hemorrhage: Biconvex, hyperdense area along the calvarium of right fronto-parietal regions. Note left occipital contusion

Subdural hematomas appear as hyperdense concavo-convex collection over the brain convexity displacing the brain parenchyma inwards (Fig. 11). An acute subdural hematoma is uniformly hyperdense while subacute hematoma shows mixed areas of hypo and hyperdensity. A chronic subdural hematoma is hypodense, almost nearing the CSF density. A subdural hematoma is concavo convex in shape and extends across the bony sutures. This is unlike the extradural hematoma which is biconvex in shape and is limited by the dural attachment to the bony sutures (Fig. 12). An extradural hematoma is generally associated with an underlying bone fracture in trauma setting.

**Fig. 13:**
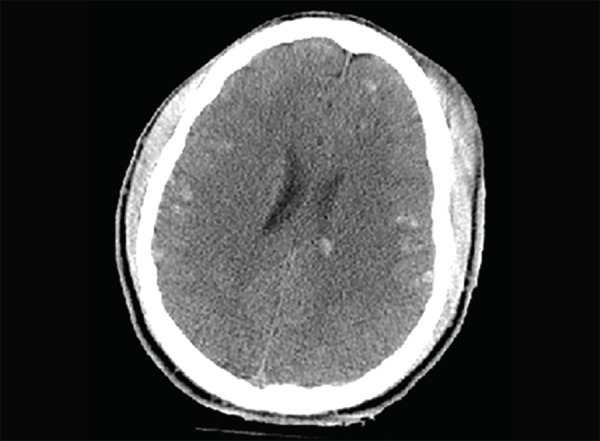
Diffuse axonal injury: multiple hemorrhagic contusions in both cerebral hemispheres, corpus callosum and diffuse cerebral edema

**Fig. 14:**
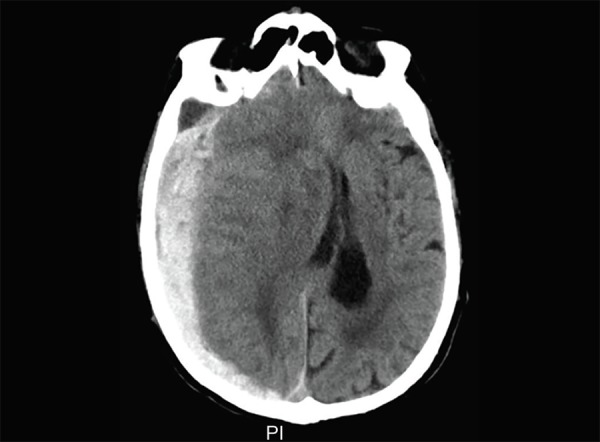
Subfalcine herniation: toward left side due to right subdural hematoma

**Fig. 15:**
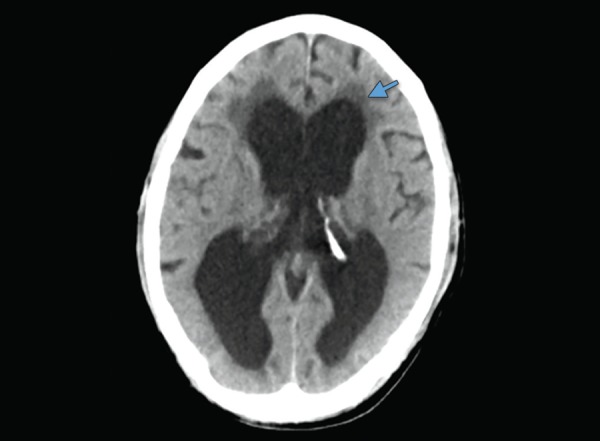
Hydrocephalus: dilated both lateral ventricles with periventricular ooze (arrow)

## TRAUMA

CT scan is the most appropriate imaging technique in acute trauma. The hemorrhagic contusions are seen as mixed of hypo and hyperdense areas on the brain surface. They are found opposite to the site of impact on the cranium- contre coup location (Fig. 12). Both intra and extra parenchymal hemorrhages are seen associated with trauma. In diffuse axonal injury (DAI), small hemorrhages are seen in the deeper brain structures and grey white matter interfaces (Fig. 13). DAI may be underappreciated on the initial CT scan, however based on clinical suspicion a follow-up CT scan will show diffuse cerebral edema and small punctate hemorrhagic foci. Diffuse cerebral edema is seen as effacement of the ventricles and cisterns. In cases of unilateral brain contusion, hematomas there can be midline shift and herniation of brain structures through the dural notches. In trans tentorial herniation, the medial temporal lobe bulges through the tentorial incisura and causes compression and rotation of midbrain effacing the ipsilateral cisterns and ventricles. Presence of herniation is an indication for surgical decompression. In subfalcine herniation, the anterior cerebral hemisphere herniates under the falx and gets displaced to the contralateral side (Fig. 14). Progressive untreated herniation leads to vascular compression and infarcts. Herniation of cerebral tonsils can be difficult to appreciate on CT scan, however can be seen as effacement of the perimedullary CSF spaces. Herniation of brain, effacement of ventricles and cisterns is an indication of significant mass effect and correlating with deteriorating patient condition requiring urgent surgical decompression.

## HYDROCEPHALUS

Dilatation of the ventricles accompanied by subendymal CSF ooze and mass effect indicates hydrocephalus (Fig. 15). Assessment of ventricular dilatation is mostly subjective, the temporal horns being the earlier to dilate. Rounding frontal horns is also a useful sign for hydrocephalus. The presents of mass effect is effaced by effacement of the cerebral sulci. The subependymal seepage of CSF is seen hypodensity along the ventricular walls in the deep brain parenchyma. Hydrocephalus in the acute care settings can be due to intraventricular hemorrhage, meningitis or intraventricular tumors.

**Fig. 16:**
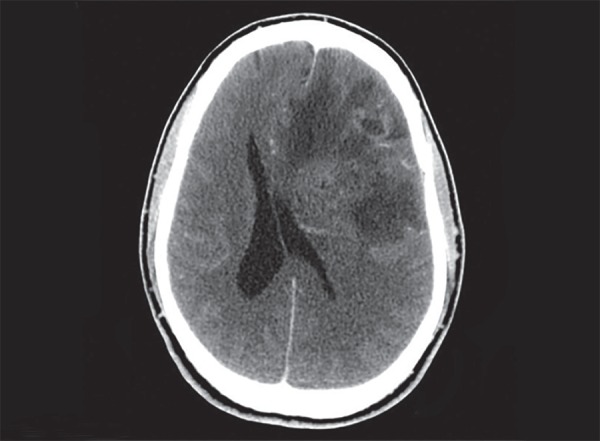
Intracranial SOL: Ill defined, heterogeneous hypodense lesion in the left fronto-temporal lobes with mass effect and midline shift towards right side

## TUMORS

Occasionally, intracranial tumors if they develop acute edema or hemorrhage may present in acute setting. Tumors are seen as focal rounded altered density lesions with displacement of adjacent structures (Fig. 16). Perifocal edema is seen as hypodensity around the solid tumor and causes mass effect and compression of ventricles with midline shift. A contrast enhanced study is required to detect enhancing component of the solid tumor. MR imaging is the most appropriate modality in diagnosing intracranial neoplasms.

## INFECTIONS

CT is relatively insensitive in diagnosing meningitis. It is usually used as a follow imaging tool to assess development of hydrocephalus or infarcts secondary to vasculitis. Occasionally a contrast enhanced CT may be done to look for presence of enhancing exudates in tubercular meningitis. However MRI is more sensitive to detect meningeal enhancement or exudates. Post contrast CT may also be done to look for presence of ring enhancing infective granulomas like tuberculomas and cysticercosis however currently, MRI has replaced CT scans in these situations.

## CONCLUSION

CT imaging is the ideal imaging technique for initial evaluation of neurocritical care patients. It is robust, reliable, widely available and relatively easy to interpret. Its ability to diagnose hemorrhage, bony fractures, brain edema and herniation in short scanning times makes it the first line imaging technique for neurological emergencies. MRI can be used as a complementary modality with its superior resolution and higher sensitivity to differentiate tissues and pathology. MRI can be used a first line in suspected focal brain lesions, hyperacute infarcts and meningitis.
